# A Multi-Step CNN-Based Estimation of Aircraft Landing Gear Angles

**DOI:** 10.3390/s21248440

**Published:** 2021-12-17

**Authors:** Fuyang Li, Zhiguo Wu, Jingyu Li, Zhitong Lai, Botong Zhao, Chen Min

**Affiliations:** 1 Changchun Institute of Optics, Fine Mechanics and Physics, Chinese Academy of Sciences, Changchun 130033, China; lifuyang19@mails.ucas.ac.cn (F.L.); 18686653384@163.com (J.L.); laizhitong17@mails.ucas.ac.cn (Z.L.); Zhaobotong19@mails.ucas.ac.cn (B.Z.); 2 University of the Chinese Academy of Sciences, Beijing 100049, China; 3 School of Computer Science, Peking University, Beijing 100091, China; minchen@stu.pku.edu.cn

**Keywords:** monocular detection, landing gear angle, CAD model, multi-step, CNN-based

## Abstract

This paper presents a method for measuring aircraft landing gear angles based on a monocular camera and the CAD aircraft model. Condition monitoring of the aircraft landing gear is a prerequisite for the safe landing of the aircraft. Traditional manual observation has an intense subjectivity. In recent years, target detection models dependent on deep learning and pose estimation methods relying on a single RGB image have made significant progress. Based on these advanced algorithms, this paper proposes a method for measuring the actual angles of landing gears in two-dimensional images. A single RGB image of an aircraft is inputted to the target detection module to obtain the key points of landing gears. The vector field network votes the key points of the fuselage after extraction and scale normalization of the pixels inside the aircraft prediction box. Knowing the pixel position of the key points and the constraints on the aircraft, the angle between the landing gear and fuselage plane can be calculated even without depth information. The vector field loss function is improved based on the distance between pixels and key points, and synthetic datasets of aircraft with different angle landing gears are created to verify the validity of the proposed algorithm. The experimental results show that the mean error of the proposed algorithm for the landing gears is less than 5 degrees on the light-varying dataset.

## 1. Introduction

The landing gear status monitoring of the aircraft aims to simultaneously detect the aircraft target to obtain landing gear angle information, which is necessary for the safe landing of the aircraft. According to flight data worldwide, more than 60% of all flight accidents occurred during the landing phase, in just a few minutes [1]. Therefore, international airports attach great importance to the tracking and monitoring of aircraft in the landing stage, and the status monitoring of landing gear is an essential aspect of aircraft attitude monitoring.

There are special inspection requirements for aircraft landing gear status monitoring. Given an image of an aircraft, the purpose of detection is to calculate the angle between the landing gears and the fuselage plain. When the measured angle is 90 degrees, the landing gears are fully down, and the plane is deemed safe to land. This goal can be divided into two aspects: to determine whether the aircraft has landing gear, and the other is to measure the actual angle between the landing gear and the fuselage plane.

The traditional landing gear observation method is to equip observers with telescopes to observe the aircraft that is about to land. The disadvantage of this method is that it is pretty dependent on the observer’s eyesight and experience judgment, and it is fatiguable and subjective when observing for a long time. Although the instrument landing system can judge the aircraft’s position, the tower still lacks direct observation to evaluate the state of the landing gears. Suppose a monitoring system can be designed to judge the status of aircraft landing gear intelligently by using aircraft images taken from the ground to give timely warning. In that case, it will save many human resources and improve the stability and objectivity of aircraft monitoring. It is of great significance to ensure the safe and stable operation of the airport.

The landing gear status monitoring of aircraft has unique application characteristics. First of all, the plane has an extensive range of motion in the flight process, and the aircraft’s speed is fast, so the scale of the aircraft varies widely in the field of the camera view. Secondly, during the aircraft’s flight, the landing gears and some key points of the plane are easily blocked due to the different angles of the camera view. Third, the cost of depth cameras and radar equipment that can obtain depth information is relatively high. The monocular camera cannot get absolute depth information of aircraft. Fourthly, pose estimation networks based on a single RGB image, such as PVNet, require the object to be a constant rigid body [2]. Since the landing gear is mobilizable, the aircraft cannot wholly be detected as a rigid body.

To solve these problems, this paper proposes a method to measure the landing gear angle of aircraft based on a monocular camera and a 3D model of aircraft without depth information. This paper estimates the key points using multi-step CNN networks, using aircraft images taken from the ground monocular camera. Then, angles of landing gears are calculated according to the constraints of the aircraft. The monocular camera is attached to a theodolite, which is used to track the plane. The aircraft is approximatively imaged at infinity relative to the camera with a telephoto fixed focal length camera. And image processing methods such as wavelet transform are used to keep the plane clear. In contrast to obtaining angles through aircraft angle sensors, the proposed method utilizes computer vision directly obtained by ground control systems. The proposed plan will work well if aircraft sensors fail to communicate with ground control systems, adding another layer of assurance for the safe landing. Additionally, the vision equipment is cheap and easy to assemble. The average landing gear angle error of this method is less than 8 degrees.

The main contributions of our work are summarized as follows:

- Object extraction and normalization modules are added to adapt to the change in aircraft target scale. The target detection module extracts the area of the aircraft, and the normalization module normalizes the area size. Then, the normalized area is inputted into the subsequent vector field regression network, voting for the key points of the fuselage. The normalization module could effectively avoid the error of the vector field network, which is caused by dramatic changes in the input image. The target detection module adopts an efficient YOLO-V4 model.
- The aircraft is divided into the landing gear and the fuselage to detect key points. The landing gears are movable, and the shape of the aircraft without the landing gear changes very little and can be regarded as a rigid body. Therefore, the aircraft as a whole can be divided into the landing gear and the two parts of the fuselage. Aircraft key points consist of landing gear key points and fuselage key points. The target detection module obtains the key points of the landing gears. The key points of the fuselage are acquired by a robust pixel-level voting network, which requires the model to be a rigid body. In addition, the distance-based coefficients are multiplied by the loss function to optimize the vector field;
- To resolve the difficulty of obtaining depth information, we propose a method to directly calculate the absolute angle between the landing gears and fuselage plane by using key positions in 2D images according to the constraints of aircraft, omitting the step of regaining 3D spatial coordinates;
- This article contributes a synthetic aircraft dataset of different camera views containing landing gears with different angles to verify the algorithm performance.

## 2. Related Works

Image enhancement is intended to be easy for machines to recognize [3]. It includes improving the contrast between the partial and whole image and improving the signal-to-noise ratio of the picture. Histogram equalization technology is used to transform the gray histogram of the original image from a relatively concentrated gray range to a uniform distribution in all gray areas [4,5,6]. Wavelet transform decomposes the image signal to enhance the low-frequency coefficient and appropriately weaken the high-frequency coefficient, and the enhanced image is obtained after reconstruction [7,8,9,10].

Target detection models and pose estimation methods based on a single RGB image have significantly been improved in recent years with the development of deep learning. Target detection based on the convolutional neural network is divided into the two-stage algorithm represented by R-CNN series with high detection accuracy [11,12,13], as well as the single-stage algorithm described by YOLO series with high precision and high speed [14,15,16]. Based on the original YOLO target detection architecture, the YOLO-V4 [17] algorithm adopts the best optimization strategy in the field of CNNs in recent years, which has varying levels of optimization for different aspects such as data processing, backbone network, network training, and so forth.

Attitude estimation based on deep learning can be divided into three parts. The RGB image fusion depth information obtained by depth camera or lidar has the highest accuracy, but it relies on expensive hardware and requires a large number of calculations [18,19,20]. The overall direct regressions based on deep learning, such as 6DVNet [21], SSD-6D [22], and so forth, lack explicit geometric constraints and have low accuracy. In addition, there are two-stage attitude estimations, such as Deepmanta [23], YOLO-6D [24], PVNet [2], and other methods. Knowing the target model, the PVNet regresses the vector field network, voting for the target key points with strong robustness. Then, the PNP algorithm is used to calculate the space pose of the target.

To represent the deformable targets and for attitude estimation, Wang et al. [25] proposed a rigid target category representation method using Normalized Object Coordinate Space (NOCS) to standardize the given target in space. NOCS only estimates the 6D posture and scale transformation of the rigid bold mark and does not have a unique representation of each part’s posture and joint connection. Li et al. [26] proposed a class-oriented representation of variable goals, ANCSH. This method divides the deformable target into different rigid parts, and the rigid parts are expressed by normalized part coordinate space.

The deformable target attitude estimation is mainly concentrated in certain areas. The primary processing method is to obtain the target instance-level information from the accurate CAD model of the deformable target. For example, Brachmann et al. [27] used the random forest to vote for the parameters of each point in the image. Pavlasek et al. expressed the attitude estimation problem of deformable objects as Markov random field. These methods are constrained by the need to model accurately. In addition, [28,29,30,31] proposed proposed ways that manipulate and interact with the target and then estimate the deformable target attitude. These methods can assess the attitude of unknown targets, but it takes a lot of time to manipulate and interact with the target, and this can only be applied to simple deformable targets. For example, Schmidt et al. [32,33] tracked deformable targets through probability inference, which requires the definition of a standard geometric structure. Daniele et al. [34] combined natural motion language information and computer vision for the attitude estimation of deformable targets, but this method requires natural motion language description as an additional mode.

In recent years, two special deformable objects, the hand and the human body, have received much attention from researchers due to their wide application. The present hand pose estimation methods are mainly divided into generation, discriminant, and mixed methods. The generation method primarily uses optimization algorithms to fit the predefined hand pose model into the given image [35,36]. The discriminant method estimates hand posture by learning the mapping relationship from the input data to the key points of the hand, such as coordinates or joint angles [37,38,39,40]. The hybrid method usually uses the optimization model in the generation method to optimize the estimation results of the discriminant method [41]. Human pose estimation can be divided into bottom-up and top-down research methods. The bottom-up approach, such as OpenPose, MultiPoseNet, and so forth, mainly predicts heat maps of different junction points and then inputs images to detect human joints and groups them according to human bodies [42,43,44]. The top-down approach sees the human body boundary frame first and predicts the position of the key nodes in the boundary frame to realize the human body pose estimations [45,46,47]. These attitude-estimation methods for particular deformable targets are only applicable to unique targets and are difficult to extend to other deformable targets.

## 3. Proposed Methods

**Figure 1:**
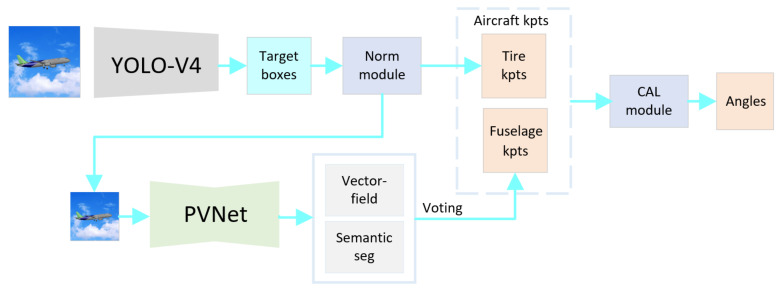
Principle of the angle estimation. Key points are abbreviated to kpts. We use YOLO-V4 to get two types of detection boxes, including the landing gear tires and the aircraft. Then the norm module gets the normalized central points of landing gear tires. At the same time, the norm module extracts the overall image inside the aircraft detection frame and normalizes the image to the PVNet network. In the PVNet network, vector fields are regressed to vote for the fuselage key points. Finally, CAL module is used to compute the landing gear angles according to key points and the aircraft model.

Figure 1 shows how the proposed method works. The angle estimation consists of two stages. The first step is to normalize the aircraft image and to detect key points of the aircraft fuselage and landing gear using the vector field network and YOLO-V4. Then, according to the aircraft’s structure and the pixel positions of key points, an algorithm is designed to calculate the angle between the landing gears and the fuselage.

YOLO-V4 network was used for the target detection. CSPDarkNet53 was used for the main feature extraction network. Feature pyramid SPP, PAN, and other structures were used to extract features [17]. Mish activation functions were used to improve accuracy and generalization performance [48]. Bilinear interpolation [49] was used in normalization, and the output size was 608 by 608 pixels. After normalization, the unstable error caused by scale variation was eliminated, and the detection accuracy was improved. Resnet18 [50] was used to obtain classification mask and the regression vector field voting for key points based on the RANSC method [2].

### 3.1. Norm Module

Firstly, the target detection module regresses out the detection frame of landing gear tires to calculate the pixel coordinates of the tires’ center points. Then, we can obtain the normalized center points according to the normalized size and the length of the object box of the aircraft. The relationship proposed in [14] is as follows:

(1) $$\left(x,y\right)=(\left(x_{max},y_{max}\right)+\left(x_{min},y_{min}\right))/2,$$

(2) $$X=\left(x-x_{min}\right)/\left(\left(x_{max}-x_{min}\right)\right)\times d,$$

(3) $$Y=\left(y-y_{min}\right)/\left(\left(y_{max}-y_{min}\right)\right)\times d,$$

where (x,y) is the pixel position of the original image, (X,Y) is the pixel position after normalization,$\left(x_{min},y_{min}\right)$ and $\left(x_{max}-y_{max}\right)$ is the upper left and lower right corner point of the detection frame, and *d* is the width of the normalized image.

In our work, we adopt a normalization model to obtain the key points of the landing gear and the normalized aircraft object.

### 3.2. Vector Field Loss Function Optimization

The target extraction module obtains the key points of the landing gear. The fuselage is treated as a rigid body, and a pixel-level vector field regression network votes for the key points of the fuselage. OpenPose [42] utilizes the vector field network to model the correspondence between key points, and PoseCNN [51] and PVNet [2] utilize it as an intermediate quantity to replace the regression of key points.

To detect the fuselage key points, the vector field regression network has two prediction objects, mask at pixel level in the target area, and the key point vector field in the target zone. The loss function proposed in [52] is composed of two parts.

(4) $$L=\lambda_{1}L_{seg}+\lambda_{2}L_{vec},$$

where $\lambda_{1}$ and $\lambda_{2}$ represent the weight of loss function. $L_{seg}$ and $L_{ver}$ represent the loss values of the two tasks of target semantic segmentation and the key point vector field regression, respectively.

In the vector field network, the binary cross entropy loss function $L_{seg}$ is used to segment aircraft and background region at the pixel level. Aircraft pixels are represented by 1 and background pixels by 0. The $L_{ver}$ use the smooth $L_{1}$ loss proposed in [52] for learning unit vectors.

(5) $$d_{k,p}=\sqrt{{\left(k_{x}-p_{x}\right)}^{2}+{\left(k_{y}-p_{y}\right)}^{2}},$$

(6) $$v\left(p\right)=\left(\frac{k_{x}-p_{x}}{d_{k,p}},\frac{k_{y}-p_{y}}{d_{k,p}}\right),$$

(7) $$L_{vec}=\sum_{k\in K}\sum_{p\in P}(smooth_{L_{1}}\left(v_{x|(p,k)}-g_{x|(p,k)}\right)+smooth_{L_{1}}\left(v_{y|(p,k)}-g_{y|(p,k)}\right),$$

where *k* is the key points. *p* is the current pixel. $d_{k,p}$ is the distance from *k* to *p*. $\left(k_{x},k_{y}\right)$ and $\left(p_{x},p_{y}\right)$ are the image coordinates of the corresponding key point and the current pixel. *K* is all the key points, *P* is the set of all instance pixels belong to the target, $v_{x|(p,k)}$,$g_{x|(p,k)}$,$v_{y|(p,k)}$,$g_{y|(p,k)}$ respectively represent the x-axis and y-axis components of the predicted values and the true values.

The vector field regression network has two prediction objects: the target area’s pixel-level mask and the key point vector field.

Depending on whether it belongs to the target pixel or not, the pixel belonging to the aircraft is represented by one, and the background is represented by zero. The classification loss, $L_{seg}$, is calculated by the cross-entropy loss function. For vector field regression mission, a Smooth L1 loss function is used to calculate the $L_{vec}$ on behalf of the sum of the loss values of normalized vectors from any key point to all pixel positions of the target.

**Figure 2:**
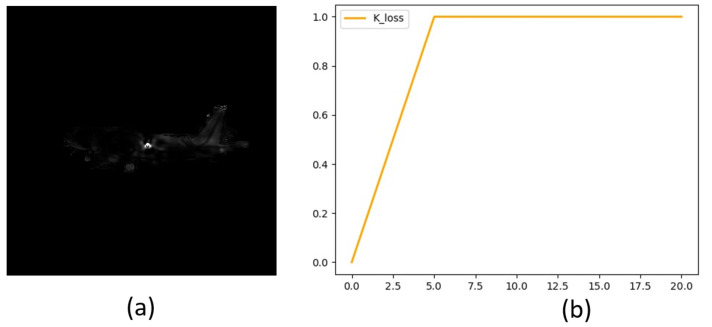
(**a**) is the visual loss value, the higher the brightness, the greater the loss value. (**b**) is the weight value K based on the distance between the pixel and key point when λ = 5. The x-coordinate is the distance and the y-coordinate is the value of K.

The final goal of the vector field regression network is to predict the coordinates of key points on the image. However, the loss function of Formula (6) loses the distance information from pixels to key points. Figure 2a shows that the pixels around the key point are closer to the key point features. The pixels around key points account for the main body of loss in the later period of training. Still, the number of pixels around key points only accounts for a small part of all pixels of aircraft targets, making it more challenging to optimize the main part of the hole pixels [53]. In this paper, an alterable vector field loss function is proposed to enhance the pixel learning of the principal amount as follows:

(8) $$L_{vec_{K}}=K\times L_{vec},$$

(9) $$whenx_{p,k}<\lambda ,K=x_{p,k}/\lambda ,when,x_{p,k}\geq \lambda ,K=1,$$

where $L_{ver}$ is the key point vector field loss function proposed in [2], K is the distance-based weight value, $x_{p,k}$ represents the distance between the current pixel point *p* and the corresponding key point *k*, and λ is a constant.

Figure 2b shows that the loss values within 5 pixels from the key point are weakened. The closer the pixel is to the key point, the smaller the value of *K*. The loss coefficient of the main part of the pixel is relatively strengthened.

### 3.3. CAL Module

As we have obtained the pixel position of the key points of the aircraft through the target detection module and vector field network, we can calculate landing gear angles without the depth information according to the constraint relations of the plane.

**Figure 3:**
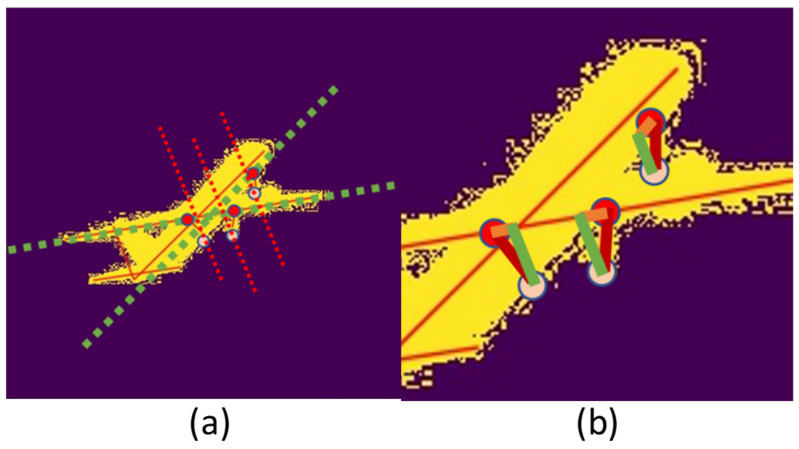
(**a**) is the mask image of the aircraft. The solid line is the key points of the fuselage, wing, and tail, the red dot is the key point of the landing gear, the pink dot is the connection point of the landing gear and the fuselage, the green dotted line is the straight line of the connection point parallel to the fuselage and wing, and the red dotted line is the straight line of the key point of the landing gear parallel to the vertical tail. (**b**) is the triangle formed by the landing gear key points, connecting points, and dotted line intersection.

Geometry has a wide range of applications in the structure [54,55,56,57]. We use the right triangle relationship and length ratio to calculate the landing gear angles. The structure of the airplane is abstracted into lines of key points as shown in Figure 3a. According to the part of the plane where the landing gear is, the landing gear to be measured is divided into the nose landing gear and the rear landing gear. The nose landing gear angle is formed by the nose landing gear and the fuselage, and the rear landing gear angle is formed by the rear landing gear and the wing belonging to the fuselage plane. Steps for calculating angles are as follows:

- Find the right triangle between the landing gear and fuselage shown in Figure 3b. Because the aircraft’s vertical tail is perpendicular to the fuselage plane, in Figure 3a, the line that is parallel to the aircraft’s vertical line is perpendicular to the fuselage plane. Considering the rotating direction of the landing gear, the vertical foot of the nose landing gear is on the fuselage line, and the vertical foot of the rear landing gear is on the wing belonging to the fuselage plane.
- Given the aircraft model, the true length in the triangle is calculated from the ratio of the length of each side to the length of the fuselage, wing, and tail respectively.
- Then the sines and cosines is used to calculate the angle θ between the landing gears and fuselage plane.

sine:

(10) $$\theta_{s}=\arcsin\left(\frac{\sqrt{{\left(x_{1}-x\right)}^{2}+{\left(y_{1}-y\right)}^{2}}}{R}\times \frac{D_{w}}{d_{w}}\right),$$

cosine:

(11) $$\theta_{c}=\arccos\left(\frac{\sqrt{{\left(x_{1}^{\prime }-x\right)}^{2}+{\left(y_{1}^{\prime }-y\right)}^{2}}}{R}\times \frac{D_{k}}{d_{k}}\right),$$

sine and cosine:

(12) $$\theta^{\prime }=\arctan\left(\frac{\sqrt{{\left(x_{1}-x\right)}^{2}+{\left(y_{1}-y\right)}^{2}}\times \frac{D_{w}}{d_{w}}}{\sqrt{{\left(x_{1}^{\prime }-x\right)}^{2}+{\left(y_{1}^{\prime }-y\right)}^{2}}\times \frac{D_{k}}{d_{k}}}\right),$$

(13) $$\theta_{z}=\sin^{2}\theta^{\prime }\times \theta_{s}+\cos^{2}\theta^{\prime }\times \theta_{c},$$

combine:

(14) $$\theta =Comb(\left[\theta_{s},\theta_{c},\theta_{z}\right]),$$

where $\left(x_{1},y_{1}\right)$ is the key point of the landing gear, $\left(x_{2},y_{2}\right)$ is the connection point between landing gear and fuselage, and (x,y) is the intersection point required. $d_{w},D_{w}$ is the pixel length of the vertical tail line in the image and the real length of the space. For the nose gear $d_{k},D_{k}$ is the pixel length of the fuselage line in the image and the absolute length of the space. For the rear landing gear, $d_{k},D_{k}$ is the pixel length of the wing line in the image and the real length of the space.

The combined method is to choose different calculation methods according to the lines’ length, based on the experience in Figure 3. Consider a combined method of sines and cosines: when the length of the fuselage or wing is greater than 340 pixels size, the cosine method is used as $\left\{\theta =\theta_{c}\right\}$. When the measurement is less than 120 pixels, the sine method is used as $\left\{\theta =\theta_{s}\right\}$. Otherwise, the sine and cosine method is used as $\left\{\theta =\theta_{z}\right\}$.

The angles of both rear landing gears are the same. The average value will be taken if both rear landing gears are in the field of view. If one of them is blocked, only the visible landing gear will be measured.

### 3.4. Synthetic Aircraft Datasets

Existing aircraft datasets are generally remote-sensing images used for target detection and classification. In contrast, few aircraft datasets are taken from the ground to the sky, and there is no dataset specifically used to obtain the attitude of the aircraft landing gear. Tobin et al. [58] proved the broad application prospect of random field sample generation through ablation experiments. Shravastava et al. [59], and Bousmalis et al. [60] trained the network by using an adversarial generation network to generate data in the target detection task and 3D pose estimation task, respectively, improving the accuracy in small sample cases. Hinterstoisser et al. [61] rendered textured CAD models from multiple perspectives to generate a large number of composite samples for training networks. The aircraft dataset with different attitude landing gears was made using an aircraft CAD model to verify the angle estimation algorithm’s effectiveness on aircraft landing gears.

**Figure 4:**
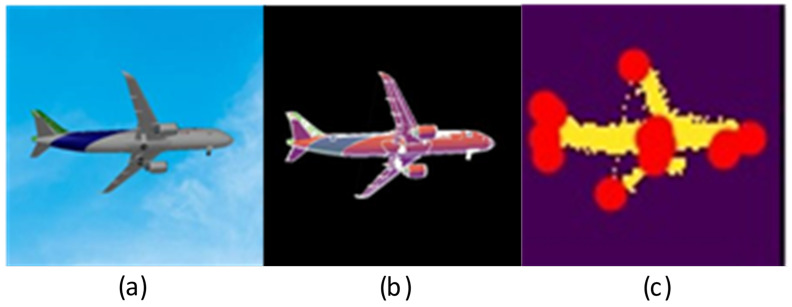
(**a**) is the original image in the dataset. (**b**) is the mask image, and (**c**) is the mask image displaying labeled key points. The dataset contains about 40,000 images.

Figure 4 shows the RGB image of the plane, the corresponding mask image, and the pixel coordinate marks of the key points. A 3D model of rendered aircraft is loaded into CAD software. Then the landing gears are rotated at different angles, as well as the aircraft is operated by rotation and translation. Different camera views are elected, and the real images and corresponding mask images are projected. At the same time, the spatial coordinates of the key points are derived, and the pixel positions of the key points are obtained according to the projection relations.

**Figure 5:**
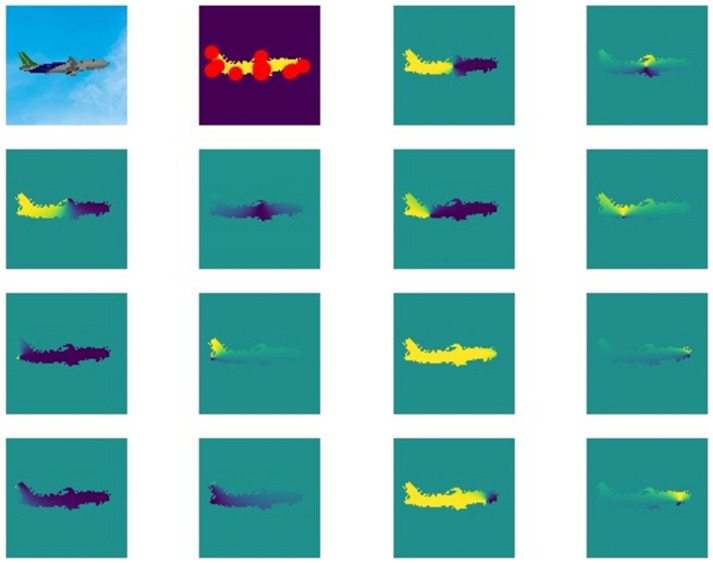
The figures above show the input data form of the vector field network model made by the dataset. In the top line, the first picture from left to right is the original image. The second picture in the top line is the mask with the labeled information of the displayed key points. The rest of the pictures are pixel vector field labels belonging to corresponding key points.

Figure 5 shows the input data form of the vector field network model made by the dataset. The normalized aircraft image and the mask of the aircraft area are used to carry out semantic segmentation of aircraft. The vector field images are used to regress the pixel-level vectors of each key point.

The key points of the aircraft are selected as corner points, and points with unique characteristics [2] that can be easily identified. A total of eight points are chosen for each image, including the endpoint of the aircraft wing, the head of the aircraft, the tail of the plane, and the center point of the aircraft. Three landing gear tires’ key points and three joining points between the landing gear and the aircraft are added to calculate the angle. The total quantity of the key points ranges up to 14. The tires’ center can be regarded as the key point, so their detection box is centrosymmetric, reducing the error of angle floating. The advantage is that the tires are apparent, and their features are easy to identify. The aircraft target detection box is a rectangle for normalization purposes. In addition, the datasets use different sky background images to increase the generalization ability of the algorithm.

## 4. Experiments

In the scale normalization module, YOLO-V4 is trained to detect the aircraft and landing gears targets. Label smoothing is set to 0.005, and training techniques were used, such as data enhancement, cyclic cosine annealing learning rate schedule, and freezing training. Considering the shielding of landing gears, it is marked for the positions of the landing gears and whether they were visible in the picture. The YOLO-V4 model is trained for about 200 rounds. For the vector field regression network, the fuselage key points is extracted by PVNet. The Adam optimizer is used, and the initial learning rate was set to 0.001, using MULTISTEP learning rate adjustment strategy. Over 200 rounds of training are taken, using an NVIDIA 3080Ti graphics card.

This paper uses the average pixel error of key points, the average angle error of landing gear, and the accuracy curve to measure the algorithm’s performance comprehensively. The landing gear to be measured is divided into the nose landing gear and the two types of the rear landing gear. The method proposed in this paper was tested on the synthetic aircraft dataset presented in Section 3.4, including parallelism and perspective. Of the dataset, 90% was randomly selected as the training set and 10% as the test set.

### 4.1. Angle Measurement Results

**Figure 6:**
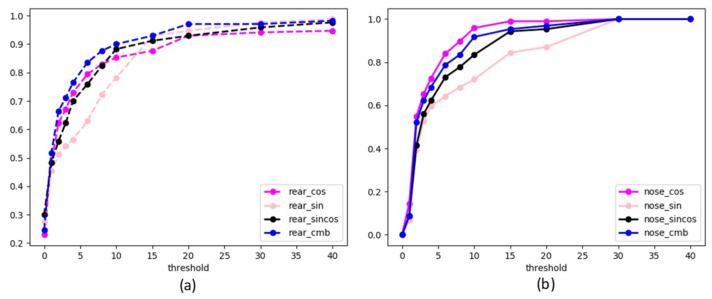
The input is the real pixel coordinates of key points, and the output is the curve of error threshold and the angle accuracy. (**a**) is the accuracy of the different methods about the rear landing gear, and (**b**) is the accuracy of the different methods about the nose landing gear.

Figure 6 and Table 1 show that when the positioning error of key points is not considered, the combined method and cosine method have better outputs. Table 1 shows that the combined process reduces the average error by 5.1 degrees of the nose landing gears, 2.5 degrees of the rear landing gears, and 1.5 degrees of the mean angle of the two types.

### 4.2. Normalized Module

Table 2 shows that the average error of the mean angle has been reduced obviously by 8.6 degrees with the normalization module, and the mean error of fuselage points is reduced by around seven pixels in size. When the error threshold is 10, the accuracy has significantly improved approximately 40% and 20% of the two types of landing gears, respectively. When the angle error of prediction increases, it becomes more difficult to correctly determine the landing gear type since the type is judged depending on the distance between the key points.

### 4.3. K Loss Function

Table 3 shows that the vector field loss function is efficient when enhancing the pixel learning of the principal part based on distance weighting. The key points pixels error of the fuselage is reduced by 1.2 pixels size, and the angle accuracy of the nose and rear landing gear increases by 0.7% and 1.1%, respectively.

### 4.4. Experiment on Different Datasets

**Figure 7:**
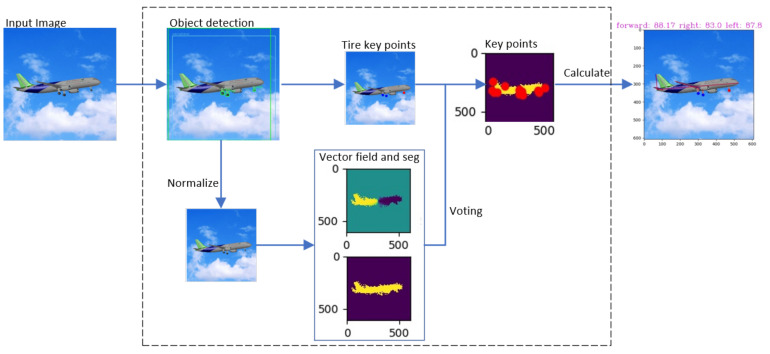
The proposed method works on datasets; predicts key points in two parts, calculates angle with no depth information.

Figure 7 shows how the algorithm works. An RGB image is inputted to detect the key points of the aircraft after normalization. Then the angle between the landing gear and the fuselage plane of the aircraft is calculated and displayed using the relationship between the key points of the aircraft.

Considering a civil airplane landing with a fixed attitude range, the angle between the vertical tail and the fuselage is close to 90 degrees during landing. The landing gear is down when the corner is 90 degrees. We subdivided the dataset into three categories. We used the data whose tail and fuselage wing angles ranged from 80 to 100 degrees as the first dataset and the landing gear of 90 degrees as the second dataset. The third kind of dataset is selected from the approximate parallel perspective of the total dataset. The projection is approximately parallel when the aircraft is far away, and the parallel projection data were also used for testing.

**Figure 8:**
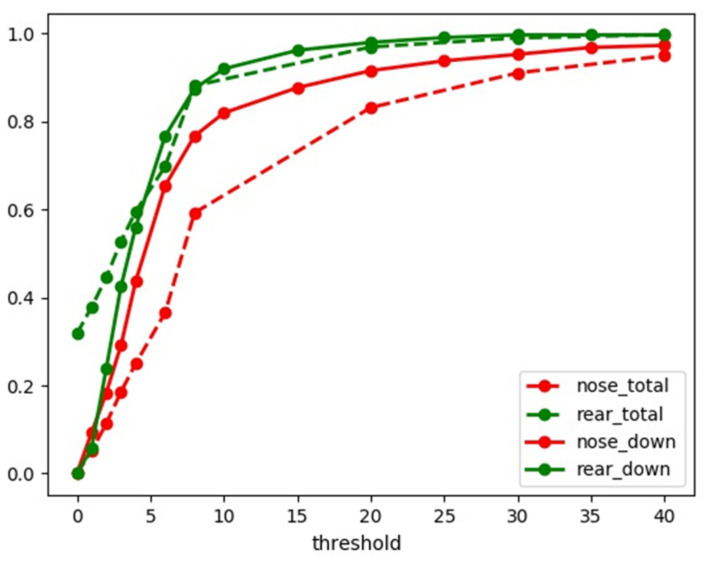
The accuracy of the total datasets and the down-undercart datasets.

Figure 8 and Table 4 show that the data within 10 degrees of tail and fuselage wing angles are much better than those obtained using a random camera view. When the landing gear is perpendicular to the fuselage plane, the mean angle error is fewer than 8 degrees. The angle accuracy of the rear landing gear is much better than that of the nose landing gear since there are two rear landing gears to obtain the average.

This experiment also shows that the proposed algorithm satisfies real-time operation requirements (>16 fps) under the conditions of the NVIDIA 3080Ti graphics card.

### 4.5. Robustness

Table 5 summarizes the results on the datasets with simulated illumination variation and motion blur of the aircraft. The CAD software created about 5000 images that simulated changing sunlight conditions with different light intensities and sun positions. Then all photos were randomly resized to 0.8–1.2 times as extensive supplements, and half of the images were arbitrarily selected for motion blur in the overall dataset. The table report in the overall dataset shows the negative impact on the performances of the model trained on the total dataset.

However, we improved model performances by training on datasets dissemination in this part. Data enhancements were used to improve the robustness, including random resizing, random rotation, and overlap. Table 5 shows that the model trained on the light-varying dataset has a better effect on the nose landing gear accuracy compared with the all-meet dataset. All images were resized to 256 × 256 pixels in the low-resolution dataset, and results show that the rear landing gear accuracy is slightly 3% lower. The accuracy ratio of the nose landing gear decreases significantly. The average angle error is within 5.6 degrees.

### 4.6. Discussion

**Figure 9:**
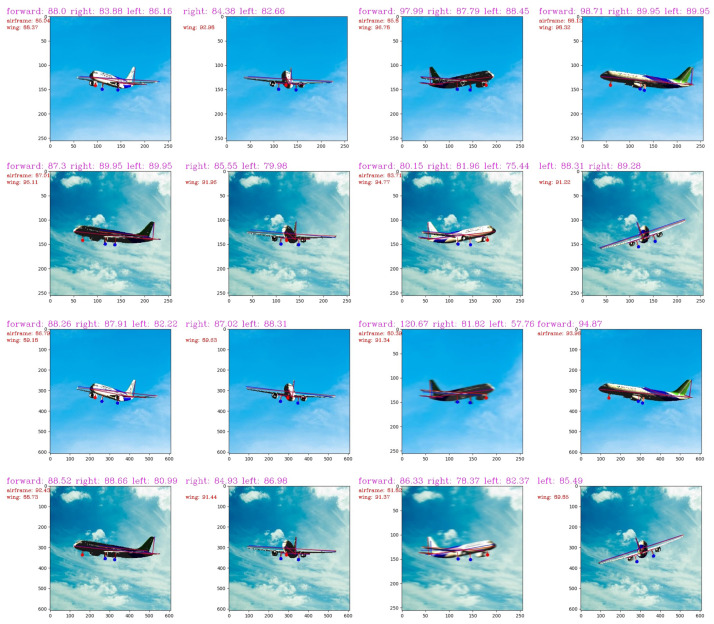
Parts of output images. The predicted angles are magenta font at the top of the graph. The number after the letter forward is for the nose landing gear angle. The number after the letter left is for the left rear landing gear angle, and the number after the letter right is for the right rear landing gear angle when facing the front. The number after the letter airframe represents the angle between the vertical tail and the airframe. The number after the letter wing indicates the angle between the vertical tail and the wing.

Figure 9 is a partial result tested on the light-varying dataset and the motion-blurry dataset. Input images are available in 608 × 608 pixels and 256 × 256 pixels. The left side of the output pictures is the angles between the vertical tail and the aircraft structure, including the airframe and the wing in RGB images. The measured landing gear angles are written at the top of the pictures.

To avoid the difficulty of angle prediction under some unique perspectives, we filter the input data of the CAL module according to the angles between the tail and the aircraft structure. Figure 9 shows the vertical tail angles to judge the accuracy of the model. When the difference between the angle and 90 degrees is greater than 10 degrees, we do not make predictions to ensure the high accuracy of the model. When the vertical tail angles are close to 90°, the algorithm is more effective. When the angle is close to 0 or 180 degrees, it becomes more challenging to determine landing gear angles correctly since they were calculated depending on the distance between the key points. Because of the fixed landing attitude of civil aviation, the vertical tail angles in the camera view will not fluctuate too much. When the steep tail angle is within 10 degrees, we can make a more accurate prediction. When the angle exceeds the range, we can also judge that the accuracy has decreased in a problematic perspective.

## 5. Conclusions

This paper proposes a multi-step CNN-based estimation of the angles between the landing gear and the fuselage plane on the monocular camera. First, the aircraft is divided into two parts to predict key points using a single RGB image. Then, the data are filtered into the CAL module to calculate the aircraft landing gear angles. Experiments have shown that the angle accuracy is improved with the target normalization module, the loss function with coefficient K, and the combined angle measurement method based on pixel coordinates of key points. In addition, we contributed a synthetic aircraft dataset containing landing gears with different angles. And we increased the robustness of illumination, making the model closer to the actual environment. We also considered wavelet transform and other methods to improve the image resolution and trained the model with high- and low-resolution images to compare the effect. The result obtained for the light-varying dataset of the proposed algorithm demonstrates that the average angle errors of the nose and rear landing gears are 6.7 and 4.5 degrees, respectively. The average angle error of total landing gears is 4.6 degrees, and the accuracy within 10 degrees is over 80%.

In future research, we will incorporate the CAL module into the end-to-end CNN, classifying rough angles and regressing pleasing results. Second, we will optimize the angle prediction under challenging perspectives. In addition, we are going to increase the variety of aircraft.

## Figures and Tables

**Table 1:** The mean error of angles and accuracy rate with different measurement methods when threshold equals 10.

Method	The Nose Error	The Rear Error	Mean Angle Error	The Nose Accuracy	The Rear Accuracy
Sine	18.7	12.4	12.2	33.8%	52.5%
Cosine	15.8	10.2	11.8	71.2%	77.5%
Sine and cosine	16.6	11.2	11.6	45.8%	62.0%
Combined method	11.5	8.7	9.1	68.9%	77.8%

**Table 2:** The mean error and accuracy rate table with or without normalization. F means false, while T indicates true.

Normalize	The Nose Angle	The Rear Angle	Mean Angle Error	Kpt Fuselage	Kpt Landing Gear	The Nose Accuracy	The Rear Accuracy
F	30.6	16.6	17.7	10.9	3.8	27.9%	58.1%
T	11.5	8.7	9.1	3.6	3.7	68.7%	78.0%

**Table 3:** This is a mean error and accuracy rate table with different loss functions. F means false, while T means true.

K Loss	The Nose Angle	The Rear Angle	Mean Angle Error	Kpt Fuselage	Kpt Landing Gear	The Nose Accuracy	The Rear Accuracy
F	11.8	8.1	8.9	4.5	3.8	66.7%	79.9%
T	11.9	7.9	8.8	3.3	3.7	67.4%	81.0%

**Table 4:** The mean error and accuracy rate for different datasets.

Datasets	The Nose Angle	The Rear Angle	Mean Angle Error	Kpt Fuselage	Kpt Landing Gear	The Nose Accuracy	The Rear Accuracy
Total	18.3	8	11.7	5	4.6	49.0%	82.2%
Parallel	11.5	8.7	9.1	3.6	3.7	68.9%	77.8%
Undercart down	12.0	8.5	10.2	2.8	3.0	70.4%	77.1%
Within 10 degrees	11.5	5.1	9.6	5	4.6	66.0%	92.1%
All meet	8.3	4.8	7.2	3	3.1	81.9%	91.9%

**Table 5:** The mean error and accuracy rate on different datasets.

Datasets	The Nose Angle	The Rear Angle	Mean Angle Error	Kpt Fuselage	Kpt Landing Gear	The Nose Accuracy	The Rear Accuracy
Overall	64.0	59.9	60.8	54.8	13.5	9.4%	5.3%
Varying light	6.7	4.5	4.6	3.7	3.3	84.8%	91.4%
Low resolution	9.1	5.2	5.6	1.9	1.4	64.9%	88.6%

## Data Availability

Not applicable.
